# Prevalence and trends in Australian adolescents’ adherence to 24-hour movement guidelines: findings from a repeated national cross-sectional survey

**DOI:** 10.1186/s12889-021-12387-z

**Published:** 2022-01-15

**Authors:** Maree Scully, Claudia Gascoyne, Melanie Wakefield, Belinda Morley

**Affiliations:** 1 Centre for Behavioural Research in Cancer, Cancer Council Victoria, 615 St Kilda Road, Melbourne, Victoria 3004 Australia; 2 Melbourne School of Psychological Sciences, The University of Melbourne, Parkville, Victoria 3010 Australia

**Keywords:** Recommendations, Physical activity, Sedentary behaviour, Screen time, Sleep, Youth

## Abstract

**Background:**

24-hour movement guidelines recommend a healthy balance of high levels of physical activity, low levels of sedentary behaviour and appropriate sleep duration each day. At present, surveillance data on how Australian adolescents are performing against these guidelines are lacking. This study aims to describe the extent to which Australian secondary school students are adhering to the physical activity, sedentary recreational screen time and sleep duration recommendations outlined in the national 24-hour movement guidelines for children and young people. It also examines whether there are socio-demographic differences in levels of compliance and if there have been significant changes in these behaviours over time.

**Methods:**

A repeated national cross-sectional survey of students in grades 8 to 11 (ages 12-17 years) was conducted in 2009-2010 (*n*=13,790), 2012-2013 (*n*=10,309) and 2018 (*n*=9,102). Students’ self-reported physical activity, screen time and sleep behaviours were assessed using validated instruments administered in schools via a web-based questionnaire.

**Results:**

In 2018, around one in four students (26%) did not meet any of the 24-hour movement guidelines, while only 2% of students met all three. Adherence to the sleep duration recommendation was highest (67%), with substantially smaller proportions of students meeting the physical activity (16%) and screen time (10%) recommendations. Differences in adherence by sex, grade level and socio-economic area were apparent. Students’ compliance with the screen time recommendation has declined over time, from 19% in 2009-2010 to 10% in 2018. However, there has been no significant change in the proportion meeting the physical activity (15% in 2009-2010 cf. 16% in 2018) and sleep duration (69% in 2009-2010 cf. 67% in 2018) recommendations. Compliance with all three guidelines has remained very low (<3%) across each survey round.

**Conclusions:**

There is considerable scope to improve Australian adolescents’ physical activity and sedentary behaviours in line with the national 24-hour movement guidelines. Policy proposals and environmental interventions, particularly those focused on replacing sedentary screen time with physical activity (e.g. promotion of active commuting to/from school), are needed to better support Australian adolescents in meeting the 24-hour movement guidelines.

## Background

In recent years, the Australian Government has released age-specific 24-hour movement guidelines that focus on achieving a healthy balance of high levels of physical activity, low levels of sedentary behaviour and appropriate sleep duration each day [1, 2]. Adapted from the evidence-based Canadian Guidelines [3], the integration of these three behaviours into a single set of guidelines reflects their co-dependency and the importance of considering movement across the whole day for optimum health. A recent systematic review concluded that meeting 24-hour movement guidelines is associated with many positive health indicators in children and young people including lower adiposity, higher fitness and better mental, social, emotional, and cardiometabolic health [4]. There is also evidence of greater health benefits when meeting more of these recommendations [5–8].

Since the adoption of 24-hour movement guidelines in Australia, there have been some efforts to document compliance among young children [9, 10]. Using accelerometer data and parent reports, these studies have found that between 15-20% of pre-schoolers are meeting all three guidelines set out for their age group, with high adherence to the physical activity and sleep duration recommendations (89-93%) and substantially lower adherence to the screen time recommendation (17-23%) [9, 10]. Prior to Australia shifting to this integrated movement behaviour model, a cross-sectional, multinational study of children aged 9 to 11 years conducted between 2011 and 2013 found that 15% of the 451 Australian participants met 24-hour movement guidelines for children and young people, defined as at least 60 minutes of moderate-to-vigorous physical activity per day, no more than two hours of recreational screen time per day and 9 to 11 hours of sleep per night (ages 5 to 13 years; 8 to 10 hours per night for ages 14 to 17 years) [6]. When each behaviour was considered independently, approximately three-quarters (76%) and just over half (55%) were adhering to the sleep and physical activity recommendations respectively (assessed using accelerometry) while around one third (35%) were adhering to the screen time recommendation (assessed using self-report). More recently, an analysis of parent-reported data from 12- to 13-year-olds participating in wave seven of the birth-cohort of the Longitudinal Study of Australian Children in 2016 found that only 2% were meeting all three of the 24-hour movement guideline recommendations and 18% were not meeting any of them [8]. Further, decreased compliance rates with the 24-hour movement guidelines have been observed among a small sample of Australian children in the transition period from grade 6 (primary school) to grade 7 (secondary school) [11]. At present, surveillance data on how older children are performing relative to these guidelines are lacking, with the only published study identified in the literature limited to data collected from grade 5 to 12 students attending a single independent school in Perth, Western Australia [12].

The most current national data on Australian adolescents’ physical activity and sedentary behaviour, collected via personal interviews in 2011-12, indicated that 8% of 13- to 17-year-olds were physically active for at least one hour each day, one in five (20%) limited their sedentary screen-based activity to two or less hours each day, and just 2% met both these recommendations [13]. There was also evidence that adherence decreased with increasing age and that a lower proportion of male adolescents were meeting the sedentary screen-based behaviour recommendation; however, differences by socio-economic group were less apparent. Findings from the Longitudinal Study of Australian Children using self-reported data obtained from the same cohort of adolescents in 2012 (12-13 years), 2014 (14-15 years) and 2016 (16-17 years) suggest that nearly three-quarters (73-74%) of 12- to 15-year-olds and approximately half (48%) of 16- to 17-year-olds are meeting the minimum sleep guidelines on school nights [14]. It is important to note, though, that these figures include adolescents who may be exceeding the upper limit of the healthy sleep duration range, with excessive sleep duration a possible sign of low sleep quality [15].

The purpose of this study was to address a knowledge gap in the literature by describing the extent to which Australian secondary school students aged 12 to 17 years are adhering to the physical activity, sedentary recreational screen time and sleep duration recommendations outlined in the national 24-hour movement guidelines, both individually and in combination in order to provide insight into particular areas of concern. It also aimed to examine whether there are socio-demographic differences in levels of compliance, which may indicate a need for targeted intervention strategies to promote healthy movement behaviours in specific adolescent sub-groups. Trends were assessed to determine whether there have been significant changes in these behaviours over the past decade among this population segment.

## Methods

### Study design and sample

Data were obtained from students participating in the National Secondary Students’ Diet and Activity (NaSSDA) Survey. First conducted in 2009-2010 (*n*=13,790 from 238 schools), and subsequently repeated in 2012-2013 (*n*=10,309 from 196 schools) and 2018 (*n*=9,102 from 104 schools), the NaSSDA Survey is a national cross-sectional study of Australian adolescents in grades 8 to 11 (ages 12 to 17 years). For each survey round, a stratified two-stage probability design was used, with schools randomly selected at the first stage of sampling and classes selected within schools at the second stage. Schools were stratified by education sector (government, Catholic and independent) and randomly selected to ensure the sample reflected distributions of sector within each Australian state and territory. Where a selected school declined to participate, they were replaced in the sample by a school with similar characteristics (e.g. education sector, location based on postcode). A school response rate of 39% was achieved in 2009-2010, with this figure decreasing to 21% in 2012-2013 and then further to 8% in 2018. Within participating schools, one class group comprising a relatively random group of students (i.e. not formed on the basis of selective criteria) was selected from each grade. Additional classes were selected where class sizes were small, consent rates were expected to be low, and/or the school did not enrol students in all grades. The student response rate was comparable in 2009-2010 (54%) and 2012-2013 (53%) before increasing to 67% in 2018.

Informed consent was obtained from both parents/carers (active in 2009-2010 and 2012-2013 and either active or passive (opt-out) in 2018 as per the requirements of each individual state and territory education authority) and all participating students. The NaSSDA Survey was administered in classrooms across Australia by an independent data collection agency. Approvals were obtained from Cancer Council Victoria’s Human Research Ethics Committee, relevant state and territory education authorities and school principals. The demographic characteristics of the student sample in each survey round are summarised in Table 1.

**Table 1:** Demographic characteristics of sample in each survey round

	2009-2010		2012-2013		2018	
	n	%	n	%	n	%
*Total*	13790	100.0	10309	100.0	9102	100.0
*Sex*						
Male	6997	50.7	5140	49.9	4363	47.9
Female	6793	49.3	5169	50.1	4739	52.1
*Grade level*						
8	4291	31.1	2894	28.1	2544	27.9
9	3794	27.5	2802	27.2	2766	30.4
10	3019	21.9	2584	25.1	2179	23.9
11	2686	19.5	2029	19.7	1613	17.7
*Socio-economic area*						
Low	4468	32.6	3637	35.3	2656	29.2
Mid	5661	41.4	3733	36.3	4173	45.8
High	3558	26.0	2925	28.4	2273	25.0
*Geographic location*						
Metropolitan	8469	61.7	5931	57.6	5943	65.3
Regional/remote	5251	38.3	4373	42.4	3159	34.7

*Note*: Percentages are rounded so may not sum to 100%. Data are unweighted

### Measures

Data on students’ dietary, physical activity, screen time and sleep behaviours were collected via a web-based, self-report questionnaire. Physical activity was assessed using a single-item adapted from the *60-minute Moderate-to-Vigorous Physical Activity (MVPA)* screening measure [16]. This measure has been shown to provide a reliable estimate of adolescents’ physical activity behaviour (intraclass correlation, 0.77) and correlates (*r*=0.40, *p*<0.001) with accelerometer data [16]. Students were asked ‘Over the past seven days, on how many days were you physically active for a total of 60 minutes or more per day?’. Those who responded with seven days were classified as meeting the 24-hour movement guideline for physical activity [2].

Sedentary recreational screen time was assessed using a subscale from the *Adolescent Sedentary Activity Questionnaire (ASAQ),* which has shown good to excellent test-retest reliability among school-aged young people [17]. Students were asked to indicate how long they spend on a usual school day, usual Saturday and usual Sunday i) watching television (including catch-up television and streaming services such as Netflix); ii) watching videos/DVDs; iii) playing video games other than on the computer (e.g. Nintendo, Xbox, PlayStation); and iv) using a computer for fun. In 2018, time spent playing on a smart phone or tablet (e.g. iPad) was also included to reflect the changing technology landscape for young people [18]. Those who accumulated two hours or less of sedentary recreational screen time on an average day ([5 x hours on usual school day + hours on usual Saturday + hours on usual Sunday] / 7) were classified as meeting the 24-hour movement guideline for screen time [2].

Sleep duration was assessed using questions adapted from the *Australian Health and Fitness Survey* [19]. Students were asked to indicate what time they usually go to bed and turn the lights out on a school night, as well as what time they usually wake up on a school day. The validity of self-reported survey estimates of sleep and wake times on school nights by adolescents has been demonstrated through comparisons with both sleep diary and actigraphy [20]. In line with the 24-hour movement guidelines for sleep [2], students aged 12 to 13 years who slept for a duration of between 9 to 11 hours on a usual school night and students aged 14 to 17 years who slept for a duration of between 8 to 10 hours on a usual school night were classified as meeting this recommendation.

Students reported their sex, grade level and residential postcode. A measure of socio-economic area was determined using the Socio-Economic Index for Areas (SEIFA) Index of Relative Socio-economic Disadvantage based on student’s residential postcode [21]. Students were categorised into low (first and second quintiles; greater disadvantage), mid (third and fourth quintiles) or high (fifth quintile; least disadvantage) socio-economic area groups. Residential postcode was also used to classify home location as metropolitan or regional/remote according to the Australian Statistical Geography Standard Remoteness Structure [22].

### Statistical analysis

Data were analysed using Stata MP version 16.1 (StataCorp, College Station, Texas) and weighted to bring each sample in line with the population of students enrolled in Australia by state, sex, grade level and education sector [23–25] and to adjust for probability of school selection and non-response. The ‘svy’ prefix command in Stata was used to account for the weighting, clustering of students within each school and stratification of the survey design. Current prevalence estimates of Australian secondary students’ adherence to none of the 24-hour movement guidelines, each individual guideline, different combinations of any two guidelines, and all three guidelines are reported overall and by sex, grade level and socio-economic area. Logistic regression analyses were conducted to examine differences in current prevalence estimates by these socio-demographic factors. Changes in overall prevalence estimates and number of recommendations being met across survey rounds (2018 vs. 2012-2013 and 2009-2010 respectively) were also assessed using logistic regression. All models controlled for sex, grade level, socio-economic area, geographic location, state/territory and education sector (government, Catholic and independent). A conservative significance level of *p*<0.01 was accepted throughout.

## Results

### Adherence to the 24-hour movement guidelines

**Fig. 1:**
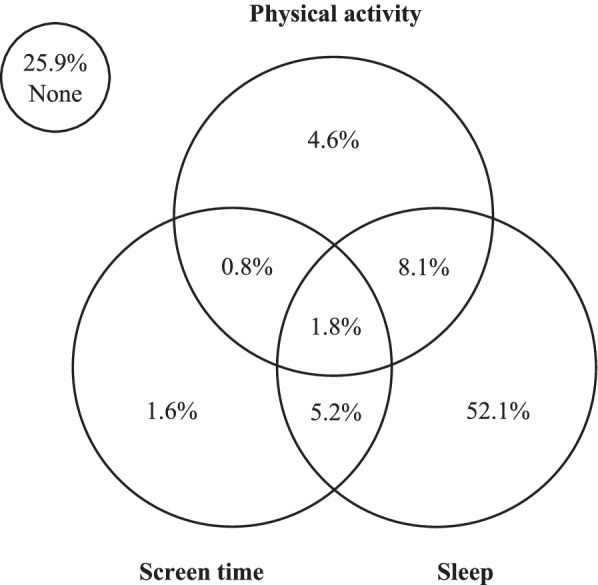
Venn diagram showing the proportions of Australian adolescents meeting 24-hour movement guideline recommendations (2018)

As shown in Table 2, around one in four students (26%) did not meet any of the 24-hour movement guidelines, while only 2% of students surveyed met all three key recommendations for physical activity, screen time and sleep duration. Adherence to the sleep duration recommendation was highest (67%), with substantially smaller proportions of students meeting the physical activity (16%) and screen time (10%) recommendations (see Fig. 1 for Venn diagram).

**Table 2:** Australian adolescents’ adherence to 24-hour movement guidelines by sex, grade level and socio-economic area (2018)

	None	Physical activity	Screen time	Sleep duration	Physical activity + screen time	Physical activity + sleep duration	Screen time + sleep duration	Physical activity + screen time + sleep duration
		% (95% CI)	% (95% CI)	% (95% CI)	% (95% CI)	% (95% CI)	% (95% CI)	% (95% CI)
*Total*	25.9 (23.2-28.8)	15.6 (13.9-17.6)	9.7 (8.4-11.2)	67.1 (64.3-69.8)	2.8 (2.1-3.6)	9.8 (8.6-11.2)	7.0 (5.9-8.3)	1.8 (1.3-2.3)
*Sex*								
Male^(ref)^	24.8 (21.9-28.0)	21.5 (19.2-23.9)	9.0 (7.2-11.1)	65.8 (62.6-68.9)	3.5 (2.6-4.7)	12.8 (11.3-14.5)	5.9 (4.5-7.5)	2.1 (1.5-2.9)
Female	27.0 (23.3-31.1)	9.4** (7.8-11.2)	10.5 (8.7-12.6)	68.5 (64.7-72.2)	1.9* (1.3-3.0)	6.7** (5.4-8.3)	8.1 (6.6-10.0)	1.4 (0.9-2.4)
*Grade level*								
8^(ref)^	24.8 (21.7-28.2)	18.7 (16.2-21.5)	14.2 (11.4-17.6)	65.3 (60.5-69.9)	3.9 (2.7-5.6)	10.9 (9.0-13.1)	9.9 (7.5-13.1)	2.4 (1.6-3.5)
9	25.8 (22.7-29.3)	17.1 (14.6-19.9)	9.7 (7.4-12.6)	68.8 (65.4-72.1)	3.2 (2.3-4.4)	12.5 (10.5-14.7)	7.4 (5.1-10.6)	2.0 (1.3-2.9)
10	24.2 (21.0-27.8)	13.9 (11.2-17.1)	7.6** (6.3-9.1)	69.8 (66.1-73.2)	2.4 (1.7-3.5)	8.5 (6.6-11.0)	5.3* (4.2-6.6)	1.7 (1.1-2.6)
11	29.2 (24.1-34.8)	12.3* (9.1-16.5)	6.7** (4.9-9.1)	64.2 (58.4-69.5)	1.3* (0.6-2.9)	7.0 (4.8-10.0)	4.8* (3.4-6.8)	0.8 (0.3-2.3)
*Socio-economic area*								
Low^(ref)^	30.0 (24.7-35.8)	16.7 (14.0-19.7)	9.4 (6.8-12.9)	61.2 (56.1-66.1)	2.7 (1.9-3.9)	9.6 (7.8-11.8)	5.9 (4.1-8.3)	1.6 (1.0-2.4)
Mid	25.3* (22.2-28.6)	13.7 (11.3-16.5)	8.9 (7.6-10.5)	68.7** (65.3-72.0)	2.3 (1.6-3.3)	8.6 (7.1-10.4)	6.8 (5.5-8.5)	1.6 (1.0-2.5)
High	22.8** (20.5-25.3)	18.2 (15.5-21.2)	11.4 (8.8-14.6)	70.2** (67.3-73.0)	3.6 (2.6-5.0)	12.4 (10.1-15.1)	8.3* (6.1-11.3)	2.3 (1.5-3.6)

* *p*<0.01; ** *p*<0.001 denotes significant difference compared to reference category ^(ref)^ after controlling for other socio-demographic factors listed in the table, geographic location, state/territory and education sector. Analyses also adjusted for the clustering of students within each school

Female students were less likely than male students to report meeting recommended levels of physical activity (OR=0.37, 95% CI: 0.30-0.45, *p*<0.001). Similarly, a lower proportion of females met the combination of physical activity and screen time recommendations (OR=0.53, 95% CI: 0.33-0.84, *p*=0.007) as well as the combination of physical activity and sleep duration recommendations (OR=0.48, 95% CI: 0.38-0.61, *p*<0.001).

Differences in compliance with the 24-hour movement guidelines by grade level were also apparent. Specifically, compared with Year 8 students, lower adherence to the screen time recommendation was reported by those in Year 10 (OR=0.50, 95% CI: 0.38-0.66, *p*<0.001) and Year 11 (OR=0.43, 95% CI: 0.28-0.68, *p*<0.001). This same pattern of results was also observed for the combination of screen time and sleep duration recommendations (Year 10: OR=0.51, 95% CI: 0.34-0.78, *p*=0.002; Year 11: OR=0.46, 95% CI: 0.27-0.77, *p*=0.003). Students in Year 11 were less likely than the youngest students to be meeting the physical activity recommendation, both individually (OR=0.63, 95% CI: 0.44-0.89, *p*=0.009) and in combination with the screen time recommendation (OR=0.34, 95% CI: 0.17-0.70, *p*=0.004).

Compared with students residing in low socio-economic areas, students residing in mid (OR=0.76, 95% CI: 0.62-0.93, *p*=0.009) and high (OR=0.57, 95% CI: 0.43-0.76, *p*<0.001) socio-economic areas were less likely to be meeting none of the 24-hour movement guidelines. Conversely, adherence to the sleep duration recommendation was higher among students residing in mid (OR=1.45, 95% CI: 1.20-1.74, *p*<0.001) and high (OR=1.79, 95% CI: 1.37-2.34, *p*<0.001) socio-economic areas. Students residing in high compared to low socio-economic areas were also more likely to be meeting the combination of screen time and sleep duration recommendations (OR=2.10, 95% CI: 1.33-3.32, *p*=0.002).

The proportion of students adhering to all three key recommendations for physical activity, screen time and sleep duration did not significantly vary according to sex, grade level or socio-economic area.

### Trends over time in compliance levels

As Table 3 indicates, students’ compliance with the physical activity recommendation has remained steady over time. There has also been no observed change in the proportion of students achieving the sleep duration recommendation since 2009-2010. However, adherence to the screen time recommendation has declined, with students less likely to be limiting their sedentary recreational screen time to no more than two hours per day in 2018 compared to 2012-2013 (OR=1.44, 95% CI: 1.23-1.69, *p*<0.001) and 2009-2010 (OR=2.17, 95% CI: 1.84-2.56, *p*<0.001). Similarly, the proportion of students meeting the combination of screen time and sleep duration recommendations was lower in 2018 than in previous survey rounds (2012-2013: OR=1.45, 95% CI: 1.21-1.75, *p*<0.001; 2009-2010: OR=2.21, 95% CI: 1.84-2.66, *p*<0.001). Compliance with all three 24-hour movement guidelines has remained very low (<3%) across each survey round. However, students were more likely to be meeting none of the 24-hour movement guidelines in 2018 compared to 2009-2010 (OR=0.82, 95% CI: 0.72-0.94, *p*=0.006).

**Table 3:** Australian adolescents’ adherence to 24-hour movement guidelines by survey round

Guideline component being met	2009-2010		2012-2013		2018	
	%	(95% CI)	%	(95% CI)	%	(95% CI)
None	22.1*	(20.7-23.6)	23.6	(22.1-25.2)	25.9	(23.3-28.7)
Physical activity	14.7	(13.5-16.1)	17.3	(16.1-18.7)	15.6	(13.9-17.5)
Screen time	19.1**	(17.5-20.9)	13.5**	(12.3-14.8)	9.7	(8.3-11.2)
Sleep duration	69.3	(67.7-70.8)	67.9	(66.0-69.7)	67.1	(64.4-69.8)
Physical activity + screen time	3.3	(2.8-3.9)	3.1	(2.5-3.7)	2.8	(2.2-3.5)
Physical activity + sleep duration	9.9	(9.0-10.9)	11.5	(10.5-12.6)	9.8	(8.7-11.1)
Screen time + sleep duration	14.3**	(12.9-15.9)	9.8**	(8.7-11.0)	7.0	(5.8-8.3)
Physical activity + screen time + sleep duration	2.3	(1.9-2.8)	2.1	(1.7-2.6)	1.8	(1.4-2.3)

* *p*<0.01; ** *p*<0.001 denotes significant difference compared to 2018 (reference category) after controlling for sex, grade level, socio-economic area, geographic location, state/territory and education sector. Analyses also adjusted for the clustering of students within each school

### Trends over time in number of 24-hour guideline recommendations being met

**Fig. 2:**
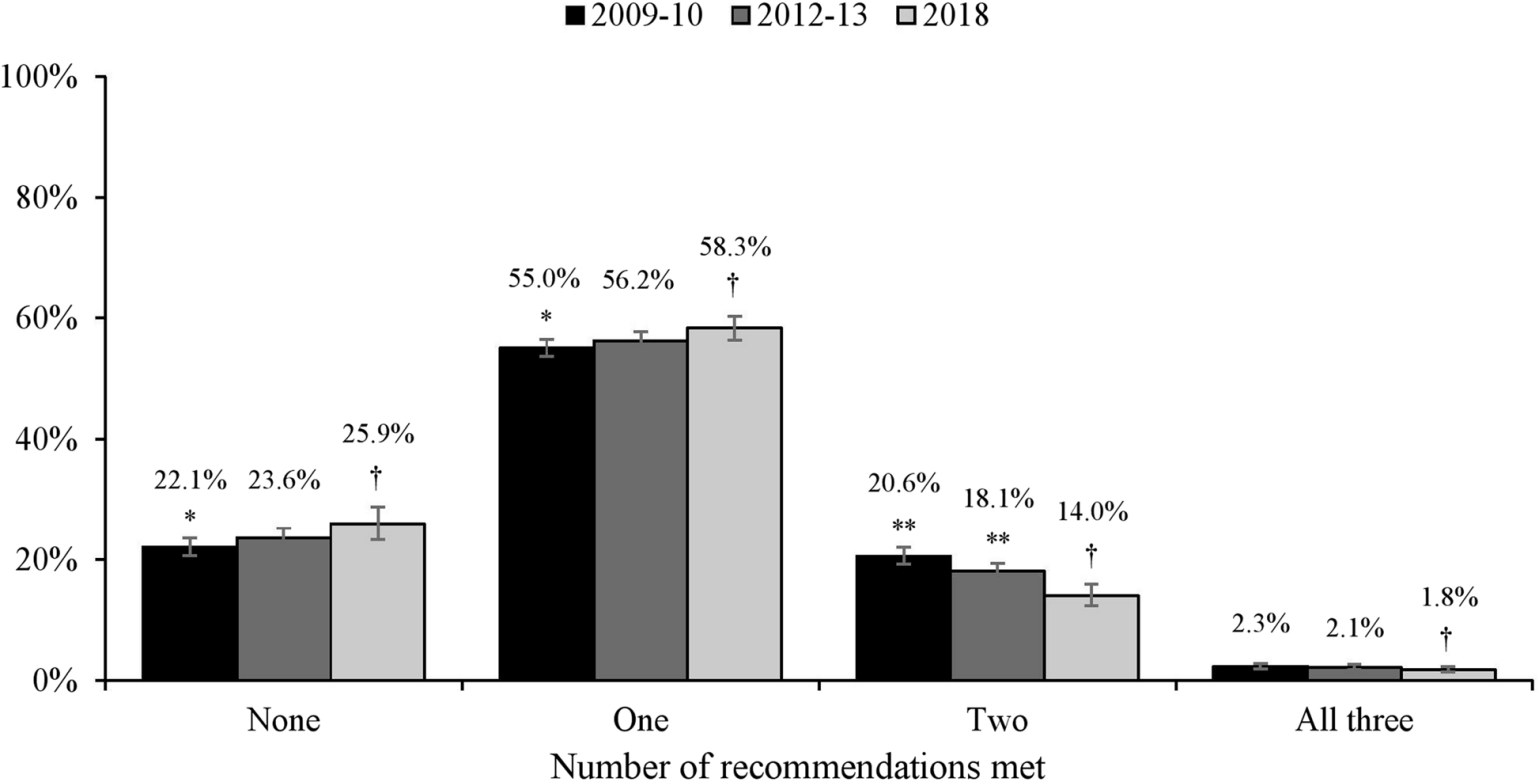
Number of 24-hour movement guideline recommendations being met by Australian adolescents by survey round. Notes: 95% confidence intervals are represented by vertical bars. * *p*<0.01; ** *p*<0.001 denotes significant difference compared to 2018 († reference category) after controlling for sex, grade level, socio-economic area, geographic location, state/territory and education sector. Analyses also adjusted for the clustering of students within each school

Changes over time in the total number of recommendations being met by students were evident (see Fig. 2). Specifically, fewer students reported meeting two of the three recommendations in 2018 compared to 2012-2013 (OR=1.35, 95% CI: 1.17-1.56, *p*<0.001) and 2009-2010 (OR=1.56, 95% CI: 1.35-1.80, *p*<0.001). Conversely, a higher proportion of students met only one of the three recommendations in 2018 compared to 2009-2010 (OR=0.88, 95% CI: 0.80-0.96, *p*=0.006) reflecting a similar increase over time observed for meeting none of the 24-hour guidelines.

## Discussion

The results of the present study provide much needed data on how Australian adolescents are performing against the national 24-hour movement guidelines. Only a very small minority (2%) of students in our sample reported meeting all three recommendations for physical activity, screen time and sleep, and this has remained consistent over time. This figure is comparable to what has been found in cross-sectional studies conducted with adolescents in Asia [26–28], South America [29], Europe [30, 31], but somewhat lower than those in North America where estimates have been slightly more inconsistent, ranging from 3% to as high as 9% [32–37]. Such variation may be due to differences between studies in how each behaviour is being measured (e.g. self-report using single- or multiple-items vs. accelerometer). Indeed, this was reflected in a cross-sectional study of 867 Brazilian adolescents that found adherence to the combined guidelines was 3% when using self-report data and just 0.2% when physical activity and sleep was measured using accelerometry (larger discrepancies between the two types of measurement were found for adherence to the individual physical activity (25% cf. 7%) and sleep (41% cf. 32%) recommendations) [29]. Consequently, future Australian studies assessing adolescent compliance with the movement guidelines using objective measures will be important to gain a more comprehensive picture of how Australian adolescents are performing. There is also likely benefit in examining potential changes in physical activity, screen time and sleep behaviours using lower thresholds than those specified in the movement guidelines (e.g. at least 60 minutes of physical activity on four or more days in the past week). This complementary approach may provide a more nuanced understanding as to whether Australian adolescents are moving closer or further away from meeting the 24-hour movement guidelines.

While around two-thirds of students in our sample reported meeting the sleep duration recommendation for their age group, compliance was considerably lower for the other two recommendations, with only 16% of students physically active for at least 60 minutes each day and just one in ten (10%) limiting their daily sedentary recreational screen time to two hours or less. The observation that adolescent females are faring worse than their male counterparts with regard to physical activity is a disparity that is evident globally [38]. Adolescent girls experience many barriers to physical activity including perceived competency, body image issues and social norms [39]. A 2017 systematic review and meta-analysis of school-based physical activity interventions targeting adolescent girls suggests that achieving behaviour change through this approach is challenging, with only a very small effect found for multicomponent or theory-based interventions [40]. Campaigns such as *This Girl Can* (run by Sport England in the UK [41] and VicHealth in the Australian state of Victoria [42]) that have been successful in inspiring women to be physically active [43, 44] may hold potential in helping to increase activity levels among female adolescents, particularly if they are well-funded, ensure frequent exposure among the target audience to the campaign messages over time and are complemented by community-based initiatives [45, 46].

Our study also indicates that older Australian adolescents are less likely to be meeting the physical activity recommendation. This is consistent with findings from longitudinal studies which describe declines in physical activity levels during adolescence [47, 48]. Although greater demands on older adolescents’ time (e.g. due to homework, part-time job) could, in part, explain this pattern of results, the concurrent finding that this group reports lower adherence to the screen time recommendation suggests that sedentary behaviour may be displacing physical activity. A further difference to emerge in our sample was that students residing in low compared to higher socio-economic areas were less likely to be meeting the sleep duration recommendation. This is in line with a recent systematic review and meta-analysis examining the association between neighbourhood socio-economic status and child (0-18 years) sleep duration, which found that sleep duration increased with socio-economic advantage [49]. While the mechanisms driving this association are not clear, it is likely to be influenced by both environmental (e.g. neighbourhood noise and safety) and parenting factors (e.g. less supervision over bedtimes). The authors of the review noted that the overall relationship between neighbourhood socio-economic status and child sleep duration was more pronounced when sleep duration was assessed objectively rather than self-reported. Thus, it is possible that our observed difference may underestimate the true effect size; however, additional studies using an objective measurement of sleep are needed to test this.

Despite previous reports of low adherence to physical activity and screen time recommendations among Australian adolescents [13, 50], our study found no evidence of positive progress over the past decade. Instead, there has been an increase in the proportion who report spending in excess of two hours per day engaging in screen-based sedentary behaviour. This trend should be interpreted with caution, though, given that smartphone and tablet use was not included when measuring screen time in earlier survey rounds. The observed decreases in the total number of recommendations being met by Australian adolescents is concerning given studies indicating better health outcomes for young people as more recommendations are achieved [5–7]. Strategies that assist in the reallocation of sedentary screen time to physical activity (e.g. promotion of active commuting to/from school [51] and participation in organised sport [52–54]) and sleep (e.g. restricting the use of electronic devices in the evening [55]) may lead to improvements in the movement behaviour profile of Australian adolescents.

Several study limitations should be acknowledged. Due to cost and feasibility issues associated with conducting large school-based surveys, information on students’ physical activity, screen time and sleep behaviours were self-reported and hence are subject to over- and under-estimations, potentially resulting in misclassification. As such, objective measurements of these behaviours are needed to validate our study findings. Further, our measure assessing sleep duration was calculated based on when students reported going to bed and turning the lights out and did not take into account varying lengths of time to get to sleep, interrupted sleep patterns, sleep quality and/or the use of electronic devices in bed after turning out the light. We also only measured sleep duration on school nights. Previous research suggests that adolescents are more likely to be meeting minimum sleep recommendations on non-school nights, possibly due to making up for inadequate sleep throughout the school week and/or being able to choose their own waking time on non-school mornings [14]. Our method of computing sedentary recreational screen time was modified in 2018 to include smartphone and tablet use which likely contributed to the temporal decrease we observed in adherence to this recommendation. Estimates of students’ total screen time also assumed that each behaviour was independent whereas it is possible for them to co-occur (e.g. playing on smartphone while watching television). Finally, the declining school response rates (39% in 2009-2010 cf. 8% in 2018) was also a limitation; however, its impact on the representativeness of our sample in each survey round was mitigated to an extent by the use of replacement schools with similar characteristics to selected schools (i.e. education sector, postcode).

## Conclusions

In conclusion, our study indicates nearly universal non-compliance among Australian adolescents with the national 24-hour movement guidelines, with considerable scope for improvement with regard to the individual physical activity and screen time recommendations. These findings underscore the need for policy proposals and environmental interventions to better support all Australian adolescents in meeting the 24-hour movement guidelines. Our study also justifies the implementation of targeted strategies to redress socio-demographic disparities, given that we observed particularly low physical activity levels among females and older students, as well as inadequate sleep among students residing in low socio-economic areas.

## Data Availability

The data used and analysed in the current study are available from the corresponding author on reasonable request.
